# A commentary on “The efficacy of peritoneal flap fixation on symptomatic lymphocele formation following robotic-assisted laparoscopic radical prostatectomy with pelvic lymph node dissection: a systematic review and meta-analysis”

**DOI:** 10.1097/JS9.0000000000002276

**Published:** 2025-01-29

**Authors:** Wujie Chen, Dihao Lv, Yinglong Huang, Mingxia Ding

**Affiliations:** a Department of Urology, The Second Affiliated Hospital of Kunming Medical University, Yunnan, China

Currently, with the development of diagnostic surgical treatments, robot-assisted laparoscopic radical prostatectomy (RARP) has gradually become the main treatment for limited prostate cancer. Since limited prostate cancer often progresses with bone metastasis or local lymph node metastasis, depending on the condition, surgeons often choose to perform RARP and pelvic lymph node dissection (PLND) or extended lymph node dissection (ePLND) simultaneously for patients who are suspected of having local invasion of prostate cancer, or who have a high risk of metastasis from lymph nodes in the preoperative stage, so as to minimize the risk of tumor residue and metastasis and achieve the best surgical outcome to minimize the risk of tumor residue and metastasis and to achieve optimal surgical benefit. However, with the implementation of PLND, especially expanded PLND (ePLND), the incidence of some postoperative related complications, especially symptomatic lymphocysts (sLC), which can manifest as lymphocysts, pain, lower urinary tract symptoms, infections, and thromboembolic disorders, etc. The incidence of sLC imposes unnecessary economic and health burdens on the patients and their families, and some drugs and surgical procedures designed to A number of drugs and surgical materials have been developed to reduce the incidence of sLC, but the results have been unsatisfactory.

In recent years, although modified peritoneal flap fixation (PFF) has not been explicitly recommended by guidelines or expert consensus for the time being, more and more studies have shown that it has a more significant preventive effect on the occurrence of sLC. Recently, we read with great enthusiasm the Su *et al*^[1]^ “publication, and we sincerely congratulate the authors for publishing an excellent systematic review and meta-analysis on the efficacy and safety of the PFF procedure for the prevention of sLC in patients with RARP combined with PLND, based on five randomized controlled studies and five observational studies involving a total of 3177 patients, and the authors concluded that the implementation of PFF significantly reduced the sLC and specific lymphocyte-associated symptomatic occurrence without affecting perioperative outcomes such as bleeding, operative time, and nonlymphocytic complications; therefore, PFF is a good option for preventing the occurrence of sLC after RARP combined with PLND. The authors’ meta-analysis of these yet-to-be- proven questions and the presentation of confirmed ideas associated with clinical practice deserve to be recognized.

Lebeis *et al*^[2]^ first reported in 2015 that PFF is now becoming one of the main means of preventing the occurrence of sLC. PFF promotes the smooth entry of exudate lymphatic fluid into the abdominal cavity and its absorption by the peritoneal surface by means of peritoneal flaps fixed to the pelvic wall or the bladder wall intraoperatively, which achieves the effect of drainage and absorption, and reduces the formation of sLC, and there is no significant difference in the occurrence of sLC between the peritoneal flap fixed to the pelvic wall and that to the peri-vesical tissues. There was no significant difference in the occurrence of sLC compared with that of the tissue around the bladder. We would like to make the following recommendations and questions regarding the use of PFF in patients with RARP combined with PLND: (1) experience in radical prostatectomy and robotic surgical techniques, especially the peritoneal suture approach and fixation position, are necessary to prevent sLC and other complications^[3]^. When performing RARP combined with PLND, the surgeon’s experience and technique will affect the incidence of sLC, so systematic and standardized training for surgeons is necessary to reduce the incidence of sLC. (2) Although ePLND can significantly improve the diagnostic accuracy of postoperative diagnosis in PCa patients, there is no direct evidence to confirm that it is therapeutically beneficial to PCa patients^[4]^. Meanwhile, ePLND acts as an independent risk factor for sLC^[5]^. At the same time, ePLND is an independent risk factor for sLC, which can increase the incidence of sLC, so we need to be careful when implementing ePLND. (3) Similarly, patients’ BMl, age, and use of anticoagulant drugs are independent risk factors for sLC^[6,7]^. Therefore, preoperative guidance on healthy diet and weight reduction for patients with high BMI may have a preventive effect on the occurrence of sLC; careful consideration of the surgical approach for patients of advanced age; and adjustment of the anticoagulant regimen for patients using anticoagulant drugs for a long period of time may have a preventive effect on the occurrence of sLC in the postoperative period, but more studies are needed to confirm the effect of sLC. (4) Routinely placing or not placing pelvic drainage tubes after surgery may have a negative effect on the incidence of sLC tubes had no effect on the incidence of sLC^[8]^. However, we suggest that active postoperative retention and drainage of pelvic fluid combined with patient symptoms, radiological or ultrasonographic examination to detect fluid accumulation in the PLND area can exclude the interference of blood or encapsulated fluid accumulation in the surgical area on the diagnosis of lymphocysts, and improve the diagnostic accuracy of sLC. (5) When RARP is performed and combined with PLND, the sequence of RARP and PLND has an effect on the incidence of postoperative sLC. (6) If sLC is complicated by thromboembolic disease, how can we prevent sLC from causing embolism again and how to develop a diagnosis and follow-up plan, in addition to symptomatic management?

Currently, there is an increasing number of studies related to postoperative complications of lymphocysts in the world, especially for diseases with anatomical location around the pelvis, such as rectal cancer, gynecological tumors (e.g. cervical cancer, endometrial cancer, vaginal cancer, etc., which are mostly treated by radical hysterectomy), prostate cancer, etc. However, the incidence of postoperative sLC in these diseases is indeed quite different, with a significantly lower incidence of sLC after radical surgery for rectal cancer than after radical hysterectomy for prostate cancer^[9]^. The incidence of sLC after radical resection of rectal cancer is significantly lower than that after radical prostatectomy and radical hysterectomy for prostate cancer^[9]^. Studies have demonstrated a significant correlation between the incidence of sLC and the number of lymph nodes resected in patients undergoing radical hysterectomy + PLND or ePLND, reaching a peak of 53.3% in patients with ≥60 lymph nodes resected^[10]^; the extent of PLND is similar to that of radical hysterectomy in the case of radical prostatectomy (e.g., RP, RARP, LRP). Rectal cancer lymph node metastasis often occurs in the rectal mesenteric lymph nodes (MLNs) and lateral lymph nodes/occlusion and internal iliac lymph nodes (LLNs), and the extent of the operation is often as follows: complete resection of the rectum, mesentery, and mesenteric lymph nodes within the mesentery^[9,11]^; the number of lymph nodes resected is less than that of radical prostatectomy and radical hysterectomy may be, and consequently, there is less disruption of the return pathway of the peripheral lymphatic vessels. The incidence of sLC is lower. In addition, pelvic lymph nodes, especially iliac paravascular lymph nodes and para-aortic lymph node dissection, are important risk factors for the development of sLC^[12,13]^, and we hypothesized that lymph node dissection next to the great vessels may lead to more severe disruption of the side branches of the lymphatic pathways, which results in more pronounced obstruction of lymphatic reflux, thus increasing the leakage of pelvic lymphatic fluid and leading to a higher incidence of sLC^[13]^, since radical rectal surgery often Since radical rectal surgery often does not involve lymph node dissection adjacent to the aorta and iliac vessels, the incidence of sLC is lower than that of radical prostatectomy and radical hysterectomy for prostate cancer. Therefore, in the surgical decision-making of patients with prostate cancer and gynecological tumors, we need to fully weigh the pros and cons and avoid unnecessary surgical enlargement, which may lead to lymph-related complications, especially sLC; for patients with gynecological tumors in whom lymph node dissection is necessary, we can use simple omentoplasty and omental fixation^[14]^ to reduce the occurrence of postoperative sLC; for patients who require RARP + PLND, we can apply PFF to reduce the incidence of postoperative sLC; for patients who require RARP + PLND, we can apply PFF to reduce the incidence of postoperative sLC for patients who need RARP + PLND, we can apply PFF to prevent the occurrence of sLC.

In conclusion, we sincerely thank Su and colleagues for their concerted efforts to take an important step forward in the study of the safety and efficacy of PFF in the classical peritoneal approach for RARP and PLND, which is an easy, safe, and highly effective surgical technique for preventing the development of sLC. In the future, we hope that more high-quality studies will be conducted to further explore the pros and cons of PFF.

## Data Availability

No other datasets were generated during and/or analyzed during the current study. All the information is available with the manuscript.
